# Predictors of self-harm and suicide in LGBT youth: The role of gender, socio-economic status, bullying and school experience

**DOI:** 10.1093/pubmed/fdab383

**Published:** 2021-11-27

**Authors:** V Jadva, A Guasp, J H Bradlow, S Bower-Brown, S Foley

**Affiliations:** London Institute for Women’s Health, University College London, 86-96 Chenies Mews, London WC1E 6HX, UK; Centre for Family Research, University of Cambridge, Free School Lane, Cambridge CB2 3RQ, UK; Stonewall, 192 St John St EC1V 4JY, UK; Stonewall, 192 St John St EC1V 4JY, UK; Centre for Family Research, University of Cambridge, Free School Lane, Cambridge CB2 3RQ, UK; Moray House School of Education and Sport, University of Edinburgh, Edinburgh, UK

**Keywords:** self-harm, suicide, LGBT, mental health

## Abstract

**Background:**

Lesbian, gay and bisexual (LGB) young people’s increased risk of self-harm, suicidal attempts and suicide compared with heterosexual youth is well established. The current study sought to examine whether these findings also apply to the trans (T) population and which factors act as additional risk or protective factors.

**Methods:**

In a national cross-sectional survey, 3713 LGBT adolescents, aged 11–19 years, reported on their own history of self-harm, suicidal ideation and suicide attempts, as well as their experiences of school and homophobic, biphobic and transphobic bullying. Logistic regressions tested the association between risk and protective factors on self-harm, suicidal ideation and suicide attempts.

**Results:**

A high proportion of the sample reported self-harm (65.3%), suicidal ideation (73.8%) and suicide attempts (25.7%). Demographic risk factors included identifying as female, non-binary or trans and being from a low-income background. Bullying and online bullying were associated with an increased risk for each outcome, and positive school experience was associated with a reduced risk for each outcome.

**Conclusions:**

Consistent with minority stress theory, the study found high rates of mental health problems within LGBT youth. Interventions focused on improving young people’s experiences in schools appear useful targets to help improve mental health outcomes.

## Introduction

The increased risk of self-harm, suicide attempts and suicide among lesbian, gay and bisexual (LGB) young people compared with heterosexual youth is well-established,^1^ yet we still know very little about which factors contribute additional risk or protection. Most studies have focused only on lesbian and gay youth.^2^^,^^3^ The few studies of trans people’s experiences report higher rates of suicidal thoughts, suicide attempts and suicide compared with cisgender people.^4^ One UK survey of trans adults reported lifetime suicidal ideation of 84% and attempted suicide of 48%.^5^ In contrast, a survey conducted in England reported lifetime rates of suicidal thoughts and attempted suicide as 20.6 and 6.7%, respectively, among adults aged >16 years.^6^ Furthermore, a wide range of identities fall under the umbrella term of trans, including non-binary and binary trans identities. It has been suggested that as non-binary individuals (who do not identify as exclusively male or female) do not fit the classic binary transition narrative, they may experience more stress than binary trans individuals (trans men and trans women).^7^ In accordance with this notion, one Spanish study found that non-binary young people experienced higher online bullying and lower levels of support from family and friends than cis or binary trans young people.^8^ However, research that distinguishes between groups under the trans umbrella is scarce.

The high prevalence of mental health difficulties within the LGB population has been explained using Meyer’s minority stress theory.^9^ This theory suggests that the stigma, prejudice and discrimination (minority stress) associated with being a sexual minority are responsible for higher rates of mental health issues within this population. The model asserts that there are distal and proximal stressors associated with being LGB. Distal stressors are external to the person, including homophobic and biphobic discrimination, prejudice and violence; proximal stressors are internal to the person, including expectation of rejection, concealment of LGB identity and internalized homophobia and biphobia. The theory also contests that protective factors exist at both the community and individual levels, including social support and individual resilience.

This model has since been extended to the trans population, due to similarities between homophobia, biphobia and transphobia,^10^ and used within LGBT youth populations. For example, a US longitudinal study found that LGBT youth with low social support experienced higher distress throughout late adolescence than those with high social support.^11^ A Canadian study of trans youth found that experiences of discrimination, harassment and violence were related to poorer mental health outcomes.^12^ In terms of protective factors, the 2004 Minnesota Student Survey, which included data from 2255 LGB students, showed that family connectedness, support from other adults and school safety acted as protective factors for suicidal risk.^13^ Similarly, an American study of LGBT young people found school connectedness acted as a protective factor for suicidal ideation.^14^

The present study aimed to examine risk and protective factors for mental health problems among a large sample of young people self-identifying as LGBT in the UK. Specifically, the study examined the role of distal variables, specifically bullying, low socioeconomic status (SES) and school experience, on suicidal ideation, suicidal attempts and self-harm. In line with minority stress theory, we hypothesized that bullying and low SES would be associated with poorer outcomes, while perceived support from schools would act as protective factors. In light of the lack of research into trans and non-binary young people, we explored whether different risk and protective factors affect each group in the same way. In the present study, respondents could select their gender from three options: ‘male’, ‘female’ or ‘prefer to use own term’. We examined differences between young people who used their own gender term (also referred to in this paper as falling under the non-binary umbrella) to those who identified as male or female. Young people were asked if they were trans and could respond as ‘yes’, ‘no’ or ‘unsure’. For the present study, young people who selected ‘yes’ to that question were defined as trans.

## Methods

### Procedures

Survey data were collected in collaboration with Stonewall, a UK-based charity that supports the rights of lesbian, gay, bisexual and trans people across Britain. The survey was promoted widely through Stonewall’s extensive networks with hundreds of schools and youth organizations throughout England, Scotland and Wales. The survey was also distributed through social media, including Stonewall’s own social media channels, and other influencers to gain as wide a reach as possible. Young people aged 11–19 years who identified as LGBT and who lived in the England, Scotland or Wales opted themselves into the survey. Data were collected between November 2016 and February 2017. The survey was available to complete online and on paper. Ethical approval for the study was obtained from (University of Cambridge Psychology Research Ethics Committee). A waiver of parental consent was granted to protect young people who may be harmed if their LGBT identity was disclosed to their parents or guardians. Contact details for support groups and services were provided to respondents alongside the questions on mental health and at the end of the survey.

### Sample

The sample consisted of 3713 adolescents (*M*_age_ = 16.42 years, standard deviation (SD) = 1.79). As illustrated in Table 1, over half of the sample identified as female (*n* = 1996, 53.8%), a third identified as male (*n* = 1241, 33.4%) and a substantial minority used their own term, hereafter referred to as non-binary, the most common being non-binary, agender and genderqueer (*n* = 467, 12.6%). In terms of trans status, 16% of the sample (*n* = 594) identified as trans.

**Table 1:** Descriptive statistics for main study variables

Measure	*n*	%
Self-harm		
Yes	2423	65.3
No	1262	34
Suicidal ideation		
Yes	2740	73.8
No	935	25.2
Suicide attempts		
Yes	955	25.7
No	2721	73.3
Gender		
Male	1241	33.4
Female	1996	53.8
Non-binary	467	12.6
Transgender		
Yes	594	16
No	2834	84
Sexuality		
Gay/lesbian	1418	38.2
Bisexual	1357	36.5
Heterosexual	49	1.3
Unsure	247	6.7
Own term	632	17
Free school meal status		
Eligible	354	9.5
Not eligible	3236	87.2
	*M* (SD)	Range
Age (years)	16.42 (1.79)	11–19
HBT bullying	1.64 (2.21)	0–11
HBT online bullying	1.00 (1.52)	0–7
Positive school experience	2.66 (0.63)	1–4

Note. HBT = Homophobic, Biphobic, Transphobic bullying scores.

With regards to sexual orientation, over a third identified as gay or lesbian (*n* = 1418, 38.2%), a similar proportion identified as bisexual (*n* = 1357, 36.5%) and a smaller number used their own term (such as pansexual or queer), were unsure or identified as heterosexual (respectively, *n* = 632, 17%; *n* = 247, 6.7%; *n* = 49, 1.3%). Participants attended schools in England, Scotland and Wales (respectively; 82%, 10.8% and 7.2%), 84.7% were White British and 9% were from Black, Asian and minority ethnic backgrounds and 9.5% were in receipt of free school meals (i.e. from low-income family backgrounds).

### Measures

#### Self-harm and suicide

Participants indicated yes or no to whether they had ever (i) deliberately harmed themselves; (ii) thought of taking their own life or (iii) tried to take their own life.

#### Demographics

Participants provided information about their age, ethnicity, gender (male, female, own term), trans identity (yes/no/unsure), sexuality (lesbian/gay, bisexual, heterosexual, unsure, own term) and free school meal status (i.e. proxy for low household income).

#### Bullying

Participants responded yes or no to whether they had experienced any of the 11 different forms of bullying for being LGB and/or T (e.g. verbal, physical and sexual assault) and any of the 8 forms of online bullying (e.g. mean/embarrassing messages/videos, threatening messages or filming/photography without consent). Two homophobic, biphobic, transphobic (HBT) bullying scores, reflecting ‘traditional’ and ‘online’ bullying, were created by summing across each item, with a high score indicating more experiences of bullying.

#### School experiences

Using a 4-point scale (1 = ‘strongly agree’ to 4 = ‘strongly disagree’), participants rated the extent to which they agreed with six statements regarding the positivity of their school experience (e.g. ‘I enjoy going to school’, ‘I feel safe in my school’, ‘I feel able to be myself at school’, ‘I worry about being bullied at school’, ‘I feel part of my school community’, ‘There is an adult at school who I can talk to about being lesbian, gay, bisexual, trans’). A mean score was created, with a high score indicating a more positive experience of school, Cronbach’s α = 0.81.

### Analytic strategy

Three sets of logistic regressions were conducted in Mplus^15^ to examine the association between self-harm, suicidal ideation and suicide attempts and risk and protective factors (including demographic, bullying and school measures). We specified a maximum likelihood estimator and report odds ratios (OR), 95% confidence intervals (CIs) and significance levels to provide an estimate of the increased likelihood of adolescents endorsing the outcome compared with adolescents with and without the specific characteristic. To avoid loss of data, we used a full information approach so that all participants who provided partial data could be included in the analysis (*n* = 49 had missing items for some positive school experience items).^16^

## Results

### Prevalence of self-harm, suicidal ideation and suicide attempts

A high proportion of the sample endorsed self-harm (65.3%, *n* = 2423), suicidal ideation (73.8%, *n* = 2423) and suicide attempts (25.7%, *n* = 955). Bullying was common; 45% of adolescents reported at least one incident of HBT bullying (e.g. physical/verbal) and 39% reported at least one incident of online HBT bullying.

### Predictors of self-harm, suicidal ideation and suicide attempts

We used logistic regression analysis to examine predictors of adolescents’ reports of self-harm, suicidal ideation and suicide attempts. Specifically, we regressed self-harm, suicidal ideation and suicide attempts on demographic measures, including female gender identity (no = 0, yes = 1), non-binary gender identity (no = 0, yes = 1), trans identity (none = 0, yes = 1), bisexual (where lesbian, gay, heterosexual, unsure and own term was the reference group = 0) and free school meal status (no = 0, yes = 1), alongside other continuous risk and protective measures, including HBT bullying, online bullying and reporting a positive school experience. We also included adolescent age as a background control measure.

As shown in Table 2, the greatest demographic risk factor for self-harm, suicide ideation or suicide attempts was reporting a trans or non-binary gender identity. Specifically, trans adolescents, compared with non-trans young people, were almost four times more likely to report self-harm, over three times more likely to report suicidal ideation and two and a half times as likely to report an attempted suicide. Compared with those who identified as male or female, adolescents who identified as non-binary were four times more likely to self-harm, twice as likely to report suicidal ideation and 20% more likely to report attempting suicide. In addition, females were at a greater risk for each outcome: a four times greater likelihood of reporting self-harm, 60% more likely to report suicidal ideation and 75% more likely to have attempted suicide. The model was re-run excluding females who identified as trans. This showed that females were still ~4× more likely to self-harm (OR 4.67), >50% more likely to report suicidal ideation (OR 1.56) and almost twice as likely to report having attempted suicide (OR 1.95). In addition, compared with their peers, adolescents who were in receipt of free school meals were 70% more likely to report attempted suicide and almost 40% more likely to report suicide ideation. While compared with gay or lesbian young people, there was a modest (25%) but increased likelihood of self-harm or suicide attempts in young people identifying as bisexual.

**Table 2:** Logistic regression results: predictors of self-harm, suicidal ideation and suicide attempts in LGBT+ youth (OR and 95% CI)

	Self-harm (OR)	Suicidal ideation (OR)	Suicide attempts (OR)
Age	1.09^***^ [1.05, 1.14]	1.11^***^ [1.07, 1.16]	1.09^***^ [1.05, 1.12]
Female gender	3.95^***^ [3.36, 4.63]	1.57^***^ [1.34, 1.85]	1.73^***^ [1.45, 2.07]
Non-binary gender	4.11^***^ [3.17, 5.32]	2.32^***^ [1.73, 3.11]	1.37^***^ [1.09, 1.72]
Trans	3.81^***^ [2.95, 4.90]	3.32^***^ [2.53, 4.37]	2.45^***^ [1.99, 2.96]
Bisexual	1.26^*^ [1.06, 1.49]	1.23 [1.04, 1.47]	1.34^*^ [1.12, 1.61]
Free school meals	1.26 [1.00, 1.60]	1.37^*^ [1.05, 1.78]	1.72^***^ [1.38, 2.13]
HBT bullying	1.17^***^ [1.12, 1.21]	1.17^***^ [1.12, 1.22]	1.20^***^ [1.16, 1.25]
HBT online bullying	1.26^***^ [1.19, 1.33]	1.27^***^ [1.19, 1.35]	1.22^***^ [1.16, 1.29]
Positive school experience	0.57^***^ [0.51, 0.64]	0.45^***^ [0.40, 0.51]	0.67^***^ [0.60, 0.76]

Note. OR = Odds Ratio [95% Confidence Intervals]; HBT = Homophobic, Biphobic, Transphobic bullying scores.

^*^ *P <* 0.05, ^**^P < 0.01, ^***^P < 0.001.

With regards to risk factors, adolescents who experienced more HBT bullying were 17% more likely to report self-harm or report suicidal ideation, and 20% more likely to report an attempted suicide (see Table 2). Slightly stronger results were found for online bullying; adolescents who experienced more online bullying were 25% more likely to report self-harm and suicide ideation and 22% more likely to report an attempted suicide.

In terms of protective factors, adolescents who reported a more positive school experience were ~40% less likely to report self-harm or attempted suicide, and 65% less likely to report suicidal ideation.

Post-hoc analyses were conducted to explore moderation effects between trans and non-binary gender identities, and risk and protective factors, in relation to mental health outcomes. Each risk (e.g. bullying and online bullying) and protective factor (e.g. positive school experience) was centred within Mplus and subsequently multiplied with trans and non-binary gender identity to create interaction terms. The additive impact of each interaction term was examined one at a time (e.g. bullying by trans) for self-harm, suicidal ideation and suicide attempts. The lack of significant results suggests that the main effects hold across the sample as a whole and do not differ according to whether adolescents identify as trans or non-binary.

## Discussion

### Main findings of this study

Our study adds to the literature on risk and protective factors for self-harm, suicidal ideation and suicide attempts among LGBT young people, in important ways. Crucially, the large sample of young people identifying as trans and non-binary allowed a comparison of these different subgroups. We found extremely high rates of self-harm, suicidal ideation and suicide attempts among trans youth compared with their non-trans peers, consistent with previous studies of trans people.^4^ Building on this, the results also extend previous findings by showing that risk and protective factors for trans young people are similar to those of LGB young people. In addition, young people who were not trans, but who used their own term to define their gender (referred to as non-binary in this paper) showed similarly high rates of self-harm (over four times more likely) as well as being twice as likely to have suicidal thoughts and more likely to have attempted suicide compared with their male or female counterparts. This is consistent with the idea that gender non-conformity may lead to greater minority stress and highlights the importance of recognizing and acknowledging non-binary gender identity to increase awareness and to improve health care.^7^

### What is already known on this topic

A previous UK study of 889 trans adults reported high rates of suicidal ideation and suicidal attempts.^5^ That study found that having a supportive environment and timely access to gender reassignment were key protective factors, while factors such as social stigma, gender dysphoria and treatment delays and fears over gender reassignment increased the risk of suicide. Many of the adults reported having had increased feelings of gender dysphoria during adolescence, due to experiencing physical changes during puberty that were unwanted.^5^ A representative US study of 31 896 youth (398 identifying as transgender) found that transgender youth were more likely to miss school, experience bullying and perceive their school negatively than non-transgender youth.^17^ A small number of studies have compared the experiences and mental health outcomes of non-binary and binary-trans youth, and preliminary findings are mixed. A Spanish study of 782 young people aged between 14 and 25 years, of whom 180 identified as transgender and 70 as non-binary, found that young people identifying as non-binary were more likely to experience online bullying and received lower levels of support from family and friends compared with cis or binary trans young people.^8^ A UK study looked at mental health outcomes in 16- to 25-year-old trans and non-binary people, and the study found that non-binary and binary participants experienced high levels of mental health problems and that binary participants reported lower life satisfaction than non-binary individuals.^18^ However, another study of 16- to 25-year-olds in the UK (331 binary trans and 57 non-binary) found that the non-binary youth experienced significantly more anxiety and depression and had significantly lower self-esteem than the binary group, although similar rates of self-harm.^19^ Studies from the general population have found significant though comparatively lower rates of self-harm. Data from the UK’s millennium Cohort Study found 7.4% of 17-year-olds had attempted suicide.^20^ This study also found 55.8% of LGB+ young people reported self-harm in the past year compared with 20.5% of heterosexual young people, and 21.7% had attempted suicide compared with 5.8% of heterosexual young people.

Peer victimization has been found to be associated with an increased risk of suicidal ideation and suicidal attempts among children and adolescents. A large body of research now shows the relationship between peer victimization and adolescent suicide, with ~20% of adolescents considering suicide.^21^^,^^22^ A meta-analysis found that children and adolescents who had been bullied were 2.2 times more likely to experience suicidal ideation and 2.6 times more likely to attempt suicide than students who had not been bullied.^23^ Our study found that young people who used a binary term to define their gender showed high rates of self-harm and suicidal ideation compared with young people identifying as male or female. This finding adds to our limited understanding on the unique experiences of non-binary/binary-trans youth. Most preliminary studies have found that non-binary youth experience worse outcomes than binary-trans youth, although Rimes et al (2017) found that binary-trans youth experienced lower life satisfaction than non-binary youth, a finding the authors suggest may be due to limited access to medical treatment for binary-trans youth. However, the findings from this study support previous suggestions that non-binary individuals may experience more minority stress and thereby experience greater mental health problems than binary trans individuals as there is less societal understanding of non-binary identities.^7^

### What this study adds

Our study examined risk and protective factors for mental health problems among LGBT young people. Notably, young people who had experienced HBT bullying—including online bullying—were more likely to report self-harm, suicidal ideation and attempted suicide, whereas protective factors, such as a having a positive experience at school, reduced the likelihood of these harmful behaviours. This finding is consistent with minority stress theory^9^ and emphasizes the importance of improving young people’s experiences in schools to reduce negative outcomes. Given that a history of mental health problems increases the likelihood of later problems, it is important to extend the developmental scope of research on LGBT mental health into young adulthood and beyond.

The finding that participants from low-income families were at greater risk of suicide ideation and suicide attempts adds to the literature on LGBT youth and demonstrates a need for greater support of LGBT young people who are experiencing financial hardship. Similar findings of increased rates of suicide have been reported more generally, with suicide risk among adults being two to three times higher in the most deprived areas of the UK compared with the most affluent.^24^ Other data have found similar trends with males working in the lowest skilled occupations having a 44% higher risk of suicide than the male national average.^25^ Among young people, the most disadvantaged 40% of the Millennium Cohort Study were almost twice as likely to attempt suicide (almost 12% compared with around 6% of young people with higher family incomes). This study however did not find differences by family income for rates of self-harm. The combination of financial hardship and being LGBT may exacerbate risk for poor outcomes. A small number of young people who identified as trans or non-binary also fell into this low-income category; however, our ability to examine outcomes for these distinct groups was limited due to sample size. Further research is warranted to test the independent contribution of factors associated with low SES, such as family stress, on explaining the increased risk of attempted suicide.

It is important for schools and colleges to be aware that teaching about LGBT people, families and relationships and tackling HBT bullying needs to be accompanied with fostering an atmosphere of inclusivity, so that all LGBT young people feel safe and enjoy being at school and college. Bullying continues to be a risk factor for poor mental health for LGBT young people, and schools as well as online platforms need to do more to reduce rates of bullying. The increased risk of poor outcomes among young people identifying as trans and non-binary suggests that schools and colleges need to proactively address the barriers these groups may face. Indeed, while two-thirds of young people in the current study reported that their school said homophobic bullying was wrong, only a third reported their school said bullying based on a person’s gender or being trans was wrong.

### Limitations of this study

This study had a number of limitations including its cross-sectional design, which meant we could not examine causality. It is important to acknowledge that the UK does not have a nationally representative survey of LGBT youth, and thus we do not know how our sample would compare with this. While existing surveys that use a random probability design can give an overall prevalence of LGBT young people (for example the Child and Adolescent Mental Health Survey, Health Survey for England), the LGBT sample is too small to use for analytical purposes. This survey uses an opt-in approach to achieve a large enough sample to enable comparison with and across LGBT groups. Finally, the use of survey methodology meant that responses were self-reported.

## Data Availability

The data underlying this article cannot be shared publicly due to ethical and privacy reasons.

## Notes

**V. Jadva,** Lecturer, Principal Research Associate

**A. Guasp,** Former Head of Research

**J. H. Bradlow,** Former Head of Policy

**S. Bower-Brown,** Research Associate

**S. Foley,** Lecturer
